# Tumor enucleation for Castleman’s disease in the pulmonary hilum: a case report

**DOI:** 10.1186/s40792-019-0652-3

**Published:** 2019-06-10

**Authors:** Masaya Aoki, Go Kamimura, Tadashi Umehara, Aya Harada Takeda, Yui Watanabe, Koki Maeda, Toshiyuki Nagata, Tsunayuki Otsuka, Masami Sato

**Affiliations:** Department of General Thoracic Surgery, Graduate School of Medical and Dental Sciences, Kagoshima University, 8-35-1 Sakuragaoka, Kagoshima, 890-8520 Japan

**Keywords:** Castleman’s disease, Hyaline vascular type, Pulmonary hilum, Intraoperative frozen section diagnosis, Tumor enucleation

## Abstract

**Background:**

The development of Castleman’s disease in the pulmonary hilum is extremely rare. Although resection of only the lesion is sufficient because of its benign nature, lobectomy or more extensive procedures performed for the pulmonary hilar tumor have been reported.

**Case presentation:**

The patient was a 15-year-old male with a tumor in the right pulmonary hilum. Endobronchial ultrasound-guided transbronchial needle aspiration was performed but no specific findings were obtained from the cytological and histological evaluation. 18F-fluorodeoxyglucose positron emission tomography showed moderate accumulation in the tumor, which suggested potential malignancy. Intraoperative frozen section diagnosis did not show any malignant findings. Thus, we performed only tumor enucleation without any lung resection. The pathological diagnosis was hyaline vascular type Castleman’s disease. No recurrence has been observed for seven years.

**Conclusion:**

Because hyaline vascular type Castleman’s disease in solitary pulmonary hilar tumor is one of the benign diseases common in young people, intraoperative frozen section diagnosis is recommended to avoid unnecessary lung resection.

## Background

Castleman’s disease (CD) is a rare disorder of lymph propagation first reported by Castleman and colleagues in 1954 from a case with chronic fever and mediastinal tumor [1]. Histologically, there are three types: hyaline vascular type (HV type), characterized by vessel hyperplasia with hyalinization; plasma cell type (PC type), characterized by plasma cell hyperplasia and cross follicles; and a mixed type with features of both types [2]. Clinically, the localized type is characterized by an enlarged lymph node centralized in one part. The multicentric type, first reported by Gaba and colleagues in 1978, shows lymph node enlargement throughout the body [3]. Treatment for localized type CD is usually surgical resection. Although resection of only the lesion is sufficient because of its benign nature, lobectomy or more extensive procedures performed for the pulmonary hilar tumor have been reported. Here, we report a case of tumor enucleation of HV type CD in the pulmonary hilum.

## Case presentation

**Fig. 1:**
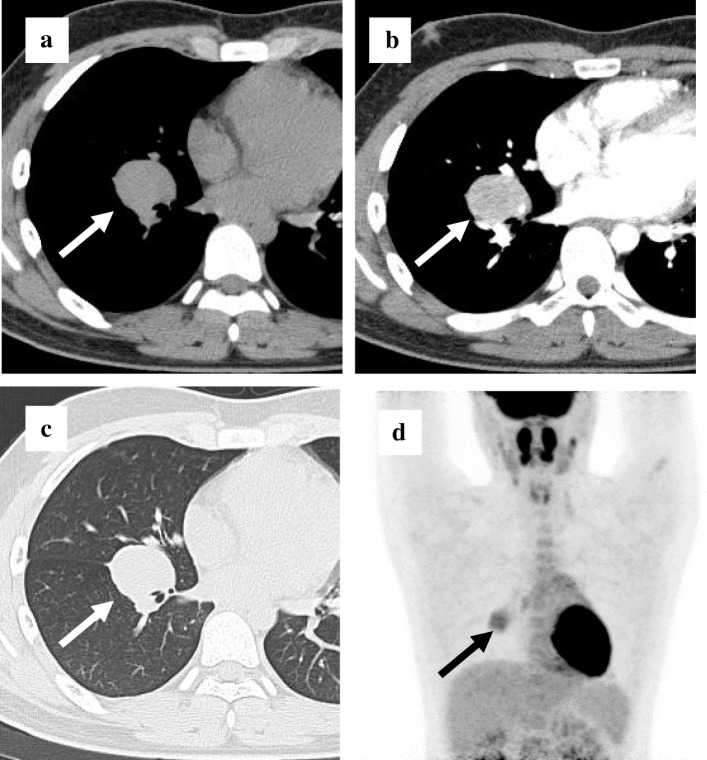
Radiological findings. **a** Non-enhanced chest CT of mediastinal condition showed a 35-mm mass in the right pulmonary hilum (arrow). **b** In enhanced chest CT of mediastinal condition, the tumor was strongly enhanced in the early phase (arrow). **c** In non-enhanced chest CT of lung field condition, abnormal findings were not found in the lung field. **d** FDG-PET scan showed accumulation only in the mass (arrow)

A 15-year-old male without any symptoms was referred to our hospital because he was noted as having an abnormal shadow on chest X-ray at a health checkup. No abnormal findings were observed on his hematological and biochemical examinations. On chest computed tomography (CT), a 40 × 33-mm wide tumor shadow with clear boundaries in the right pulmonary hilar area was found. The tumor was strongly enhanced in the early phase. Abnormal findings were not found in the lung field and mediastinum (Fig. 1a–c). Bronchoscopic examination was performed under topical anesthesia. The lateral segment of the lower lobe of the right bronchus was narrowed by compression of the tumor although the endobronchial mucosa was intact. Endobronchial ultrasound-guided transbronchial needle aspiration (EBUS-TBNA) was performed but no specific findings were obtained from the cytological and histological evaluation. However, the patient was admitted to our department for surgery because 18F-fluorodeoxyglucose positron emission tomography (FDG-PET) showed abnormal accumulation in only the tumor; SUV (standard uptake value) max was 4.4 (Fig. 1d), which suggested potential malignancy. Due to the possibility of a malignant tumor, right middle and lower lobectomy was necessary due to its localization, and depending on the intraoperative findings, it was also necessary to perform right pneumonectomy. We informed the patient and his mother of this before surgery and obtained their consent. However, from the imaging morphology of the tumor and lack of evidence of malignancy in EBUS-TBNA, we also kept in mind before surgery the possibility of benign tumors including Castleman’s disease. We decided to make a final decision on the procedure based on the findings of the intraoperative macroscopic findings and the intraoperative frozen section diagnosis.

**Fig. 2.**
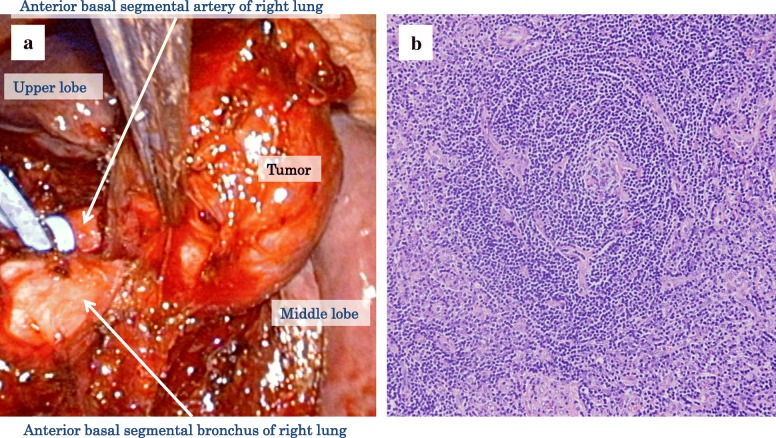
Operative findings and histological findings. **a** The tumor existed between middle and lower lobe of the right lung. Because no tumor invasion into the pulmonary artery and/or lung parenchyma were found, only the tumor enucleation was carried out. **b** There were clearly hyalinizing concentric fibrotic nests around the lymph follicle and vessel hyperplasia on the hyalinizing wall. There were almost no plasma cells outside the follicle. Histological diagnosis was HV type CD

On operative findings, the tumor existed between the middle and lower lobes of the right lung with no pleural involvement. The interlobar pulmonary artery was revealed on the back side of the tumor. We performed 18Ga needle biopsy for intraoperative frozen section diagnosis, which showed only chronic inflammation findings. Moreover, macroscopically, no tumor invasion into the pulmonary vessels, bronchi, and lung parenchyma was found. Therefore, only the tumor enucleation was performed (Fig. 2a). An intraoperative frozen section diagnosis of the removed tumor found suspected Castleman’s disease. Therefore, we decided not to do further resection.

The extirpated specimen was a 30 × 23-mm smooth and well-encapsulated tumor. Histologically, the lesion had a fibrous capsule with clear boundaries consisting of lymph follicle hyperplasia. There were clearly hyalinizing concentric fibrotic nests around the lymph follicle and vessel hyperplasia on the hyalinizing wall. There were almost no plasma cells outside the follicle. Histological diagnosis was that of HV type CD (Fig. 2b). The postoperative course was good, there were no complications, and he was discharged home on the fifth postoperative day. The patient is now under follow-up observation with no recurrence seven years after the operation.

HV type CD is common in young people. The most common sites of this disease are in the cervix, mediastinum, abdomen, and retroperitoneum [4], but it is very rare in the pulmonary hilum. HV type CD is usually discovered by chance in regular health checkups with almost no accompanying specific symptoms. In radiological findings, enhanced CT shows a contrasted tumor with clear boundaries in general, especially in the HV type. High contrast is promptly recommended for vessel hyperplasia with hyalinizing inside the tumor [5]. In addition, some reports recently found that FDG-PET sometimes shows light-to-moderate accumulation [6–8]. However, in any case, the findings are not specific for CD, making it very difficult to reach the diagnosis by radiological findings alone. Moreover, from the reports so far, definitive diagnosis by preoperative biopsy seems difficult [9–12]. Similarly, the preoperative diagnosis of our case could not be made because of insufficient biopsy specimens for the pulmonary hilar mass.

Keller et al. retrospectively examined six localized HV type CD patients who received partial resection, biopsy, or observation alone. They reported that disease progression was noted at four years after surgery in one patient and, in another patient, complete resection was performed eight years after an initial biopsy and observation following the onset of symptoms [2]. Moreover, Biçakçioğlu et al. reported that 17 of the 19 CD cases, including seven cases in the pulmonary hilum, in which surgery was performed were localized, and that 15 cases in which complete resection was performed had no recurrence [12]. Therefore, the recommended treatment strategy for localized HV type CD is complete resection. We found ten cases of resection of HV type CD in the pulmonary hilum in which detailed clinical information including surgical procedure could be obtained from the articles searched with the terms: Castleman’s disease AND pulmonary AND surgery in PubMed (Table 1) [9–11, 13–19]. One case reported by Luo et al. was described as “whole resection” and the details were unknown about surgical procedure [11]. Lobectomy or more extensive surgery was performed in seven of these nine cases. In only one of seven cases was intraoperative frozen section diagnosis performed [10]. In three of six cases, lobectomy was performed because malignancy of the tumor could not be ruled out [13, 14, 19]. Moreover, in the other case, there was no mention in the article why lobectomy was performed [17]. If intraoperative frozen section diagnosis was performed on these four cases with benign diseases as in this present case of CD, it may have been possible to select a procedure that would have preserved pulmonary function. Tumor enucleation was performed in two cases. In one of two cases, intraoperative frozen section diagnosis was performed [9]. We performed only tumor enucleation considering both the localized FDG-PET accumulation and the intraoperative frozen section diagnosis without major bleeding to carefully separate the tumor from surrounding tissue. The patient is now under follow-up observation with no recurrence seven years after the operation.

**Table 1:** Operative procedures and intraoperative biopsies in previously reported cases

Author	Age	Sex	Preoperative biopsy	Intraoperative biopsy	Procedure	Cause of lobectomy or more extensive surgery
Yeh	42	M	Not done	Not done	LUL	Potential malignancy
Tokunaga	23	F	Not done	Not done	LLL + MLND	Potential malignancy
Racil	23	F	Not done	Not done	Pneumonectomy	Bleeding at intraoperative biopsy
Wang	27	M	Not done	Not done	Enucleation	–
Gunluogle	29	M	TBNA: no specific findings	Benign	Enucleation	–
Ota	19	M	Not done	Not done	RML	Not mentioned
Nadir	28	F	Not done	Not done	RUL + MLND	Adhesion and hypervascularity
Liu	32	M	Not done	Not done	LUL + MLND	Potential malignancy
Haager	24	M	EBUS-TBNA: no specific findings	Lymphoma or carcinoid	RML	Not mentioned
Luo	40	F	TBNA: chronic inflammation	Not mentioned	Whole resection	–
Present case	16	M	EBUS-TBNA: no specific findings	Chronic inflammation	Enucleation	–

*EBUS* endobronchial ultrasound-guided, *TBNA* transbronchial needle aspiration, *LUL* left upper lobectomy, *LLL* left lower lobectomy, *RML* right middle lobectomy, *RUL* right upper lobectomy, *MLND* mediastinal lymph node dissection

``

## Conclusion

In young patients with isolated solitary pulmonary hilar tumor that does not lead to a diagnosis before surgery, intraoperative frozen section diagnosis should be performed to consider the possibility of CD. If malignant findings are not recognized in intraoperative frozen section diagnosis, the choice of procedures that preserve pulmonary function as much as possible is desirable because CD is a benign disease commonly seen in young people. However, an accumulation of cases with such localized HV type CD in the pulmonary hilum will be required to determine whether surgical mode of only tumor enucleation with long follow-up term is medically satisfactory.

## Data Availability

The datasets supporting the conclusions of this article are included within the article.

## Abbreviations

CD: Castleman’s disease
CT: Computed tomography
EBUS-TBNA: Endobronchial ultrasound-guided transbronchial needle aspiration
FDG-PET: 18F-fluorodeoxyglucose positron emission tomography
HV type: Hyaline vascular type
LLL: Left lower lobectomy
LUL: Left upper lobectomy
MLND: Mediastinal lymph node dissection
PC type: Plasma cell type
RML: Right middle lobectomy
RUL: Right upper lobectomy
SUV: Standard uptake value
